# PEG–Ursolic Acid Conjugate: Synthesis and *In Vitro* Release Studies

**DOI:** 10.3797/scipharm.1309-17

**Published:** 2014-04-06

**Authors:** Marina Zacchigna, Francesca Cateni, Sara Drioli, Giuseppe Procida, Tiziano Altieri

**Affiliations:** 1 Department of Chemical and Pharmaceutical Sciences, University of Trieste, Piazzale Europa 1, 34127 Trieste, Italy.; 2 School of Advanced Studies ”G. D’Annunzio”, University of Chieti-Pescara, Via dei Vestini 31, 66100 Chieti Scalo, Chieti, Italy.

**Keywords:** Ursolic acid, Monomethoxypoly(ethylene glycol), Conjugation, Solubility

## Abstract

A highly water-soluble macromolecular compound of ursolic acid with monomethoxypoly(ethylene glycol) (mPEG) was prepared. The physicochemical properties and stabilities under different conditions were investigated. By PEG conjugation, greatly increased water solubility was obtained, and the results showed that this conjugate was a potential prodrug for the oral delivery of ursolic acid.

## Introduction

Ursolic acid (UA) is a pentacyclic triterpene acid widely distributed in the plant kingdom, and has recently attracted much attention because of its various interesting biological activities. Ursolic acid has been used in the formulas of anti-inflammatory preparations, in the formulas of treating liver fibrosis preparations, as well as in combined chemotherapeutics for certain tumors. The interactions of these mixtures could occur chemically and biologically to produce more desired effects. Research regarding UA and the strictly correlated isomer oleanolic acid (OA) is important in the future for fully understanding these naturally occurring compounds in the treatment of various diseases [1].

In our preceding work, a simple, rapid, and isocratic HPLC method was developed and validated for the quantitative determination of the two isomeric triterpenic acids, oleanolic and ursolic acid in *Plantago major* [2].

**Fig. 1:**
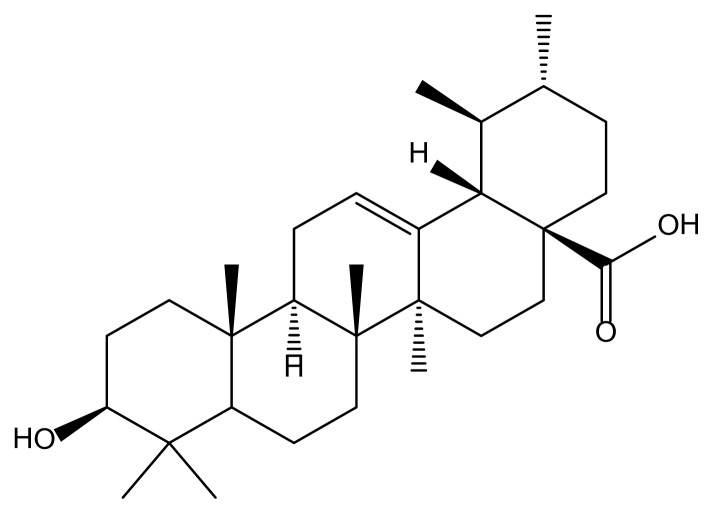
Chemical structure

UA presents a very hydrophobic structure due to its pentacyclic hydrocarbon skeleton as shown in Fig. 1, so there are many difficulties in handling the compound in biological experiments because of its extremely low solubility, especially in aqueous systems.

It is well-known that drug efficacy can be severely limited by poor aqueous solubility. It is also known that side effects of some drugs are the result of their poor solubility. Moreover, solubility problems are frequently encountered in the preparations of pharmaceutical dosage forms. Water solubility of ursolic acid is limited, thus limiting its bioavailability in the body.

Both pharmacokinetic and pharmacodynamic considerations are equally important in increasing the bioavailability and biological effects of UA.

Efforts have been made to improve the water solubility of UA with chemically modified derivatives, including a non-covalent complex with hydrophilic cyclodextrins, as well as the use of nanosuspensions [3], or the preparation of surfactant solutions and PEGs, but only moderate solubilizing capacity for UA [4] has been obtained with the methods reported. Nevertheless, there are several techniques to promote the solubility of water-insoluble drugs in aqueous systems.

Among them, the most common method is the covalent attachment of molecules to poly(ethylene glycol) (PEG). PEG is the most commonly used nonionic polymer in the field of polymer-based drug delivery [5]. Due to its high aqueous solubility, PEG polymer is considered as a versatile candidate for prodrug conjugation. PEGylation, the covalent attachment of PEG to molecules of interest, has become a well-established prodrug delivery system [6]. The remarkable properties of the biologically inert (biocompatible) PEG polymer derive from its hydrophilicity and flexibility. PEG presents particular properties such as i) lack of immunogenicity, antigenicity, and toxicity; ii) high solubility in water and in many organic solvents; iii) approval by the FDA for human use; iv) high hydratation and flexibility of the chain, which is at the basis of the protein rejection properties; and v) elimination by a combination of renal and hepatic pathways, thus making it ideal to employ in pharmaceutical applications [7]. The most-used PEGs for prodrug modification are either monomethoxy PEG or dihydroxyl PEG. Successful conjugation of PEG with biomolecules depends upon the chemical structure, molecular weight, steric hindrance, and reactivity of the biomolecule as well as the polymer. In order to synthesize a bioconjugate, both chemical entities (i.e., the bioactive as well as the polymer) need to possess a reactive or functional group such as –COOH, –OH, –SH, or –NH_2_.

In this paper, the solubility of mPEG-UA was investigated in search of a better solubilizing system for this compound.

## Results and Discussion

Ursolic acid (UA) is a triterpene acid, widespread in the plant kingdom, and has recently attracted attention for its diverse and interesting biological activities. The water solubility of ursolic acid is limited, as far as the use of the compound both *in vitro* and *in vivo* biological experiments. Macromolecular systems have been used to solubilize apolar solutes in aqueous systems. PEG is the most common polymer used for the conjugation. The reasons for PEGylation are increased solubility in water, controlled permeability through biological barriers, longevity in the blood stream, and controlled release [7]. In this paper, PEG was used to covalently modify ursolic acid.

### Synthesis of mPEG_5000_-O-CO-NH-CH_2_-CH_2_-CH_2_-NH-succ-UA (mPEG-UA)

mPEG-UA conjugate was synthesized in order to study the role of PEG conjugation as a method for improving the solubilization of UA. An ester bond link UA to succinic acid and the corresponding UA succinate at the 3 position was successively conjugated to mPEG-NH_2_ through the formation of an amide bond. Esters with an electron-withdrawing substituent (as the alkoxy group) in the α-position proved to be especially effective in the design of the prodrugs since they allowed the enzymatic hydrolysis of this bond, thus making possible the release of the alcohol moiety in a continuous and effective manner [7]. Our attention has focused to the derivatization at the 3-OH position of ursolic acid since the steric hindrance of the 28-COOH group led to a low yield of the adduct. The succinoylation of ursolic acid yielded a dicarboxylic derivative. Only the succinoyl-carboxylate at the 3 position of ursolic acid was reactive.

**Fig. 2:**
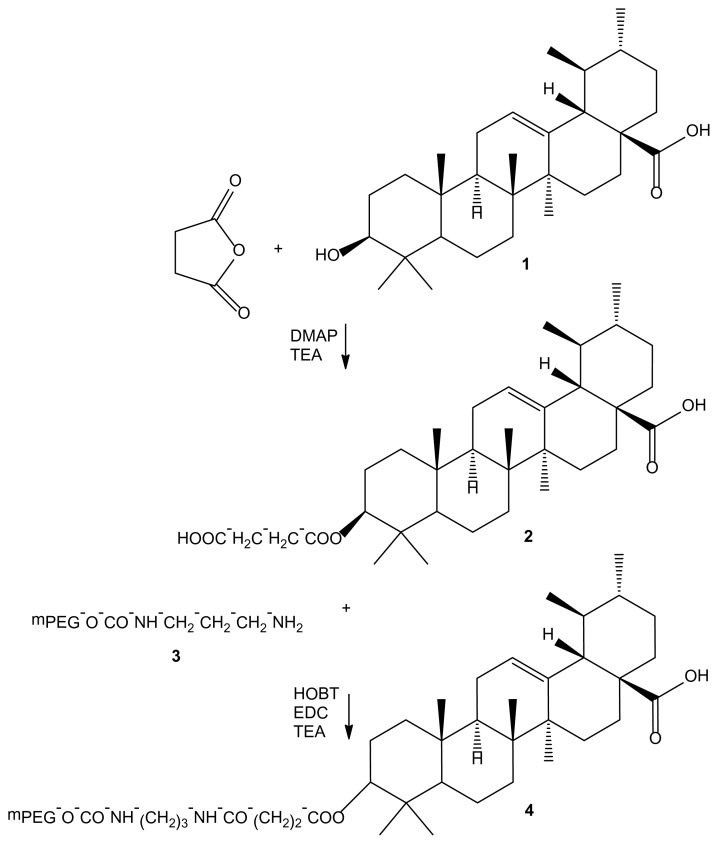
Synthesis of mPEG-UA conjugate

For the synthesis of UA prodrug, mPEG_5000_-linker-NH_2_ was used. Ursolic acid derivatization allowed the introduction of a new functional group suitable for conjugation to the polymer, through a spacer between the latter and the bioactive agent. This spacer moiety, by distancing the drug molecule from the polymeric backbone, also reduced the interaction of the latter with the drug receptor. The succinyl derivate of UA was prepared by the reaction with succinic anhydride in THF solution using DMAP and TEA as catalysts. The TEA, a strong acid scavenger base, increased the efficiency up to 50%. The attachment of UA-succinate to the aminated polymer was performed by means of HOBT and EDC as coupling agents and TEA with a satisfactory yield (95%) (Figure 2).

^1^H NMR analysis was useful in providing information for the structural elucidation of the synthesized drug-polymer conjugate. The ^1^H NMR spectrum of the adduct confirmed the presence of the methylene protons, ascribed to the PEG backbone (δ = 3.36–3.75 ppm), together with a broad singlet due to the presence of the NH proton at 7.90 ppm; additionally, the signal due to the H-3 proton of UA was observed at δ = 3.86 ppm.

The percentage of the conjugated drug was evaluated by alkaline hydrolysis followed by HPLC analysis and estimated to be near 100% (w/w). The absence of any free drug was also verified.

### Release Studies

In the synthesized adduct, the UA succinate molecule was linked by an ester bond since it was very likely that the cleavage of this bond could occur in physiological medium, thus releasing the UA. In order to gain some preliminary information about the potential use of mPEG-UA as a drug delivery system, it was subjected to hydrolysis at 37±0.1°C in buffer solutions, at two different pH, simulated gastric juice, pH 1.2, and extracellular fluids, pH 7.4, in the presence of esterase and in human plasma. Samples were regularly taken out of the incubation mixture and the percentage of released UA was quantified by HPLC.

**Fig. 3:**
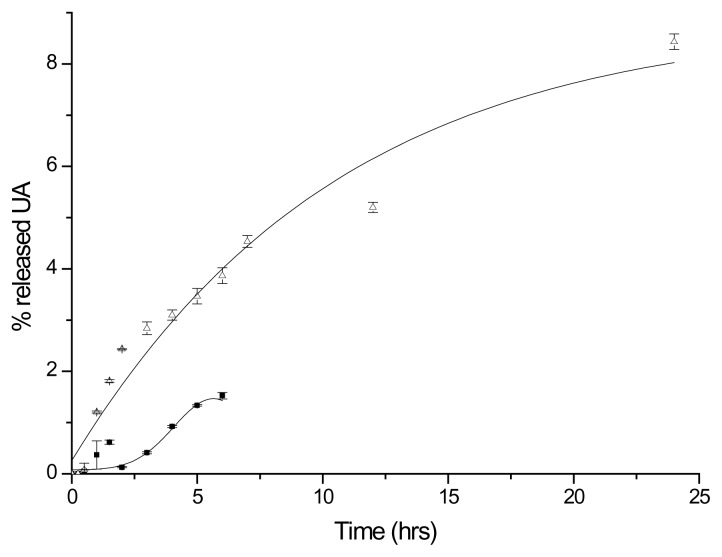
Release of UA from mPEG-UA in buffer solutions, at 37±0.1°C: ■ pH 1.2, 0.2 M HCl, NaCl, and glycine, and △ pH 7.4, 0.1 M phosphate. Each experiment was carried out in triplicate and expressed as the mean value ± S.D.

The amount of UA released from the polymeric conjugate at pH 7.4 in phosphate buffer solution was 8% over 24 h. In the buffer solution at pH 1.2, less than 1.5% of UA was released over 6 h (Figure 3). The conjugate therefore was almost hydrolytically stable both in acidic buffer at pH 1.2 and in physiological buffer at pH 7.4.

**Fig. 4:**
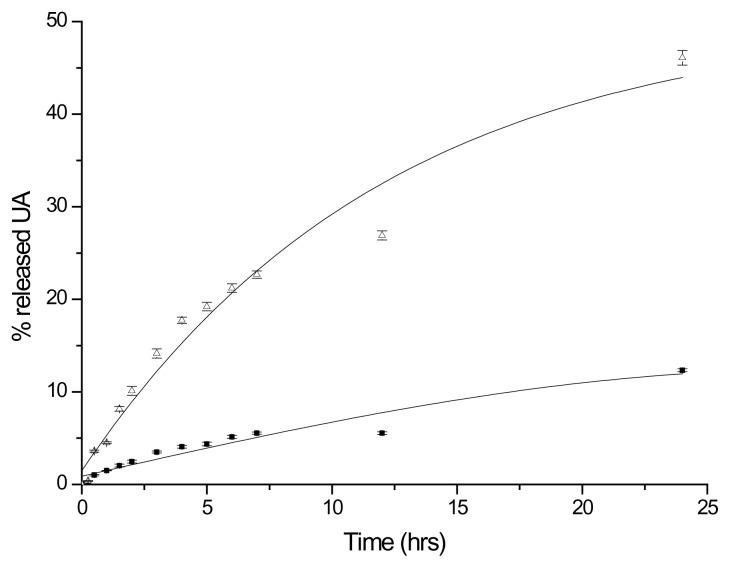
Release of UA from mPEG-UA in buffer solutions, at 37±0.1°C: ■ pH 8, 0.08 M Tris 0.1 M CaCl_2_, and △ pH 8, 0.08 M Tris 0.1 M CaCl_2_, and porcine esterase solution. Each experiment was carried out in triplicate and expressed as the mean value ± S.D.

Since the capacity of porcine esterase to catalyze the hydrolysis of ester bonds is well-known [7], the possibility that mPEG-UA may be a good substrate for this enzyme (in which drug molecules are linked to a polymeric backbone by ester linkages, via a succinic spacer) was therefore evaluated. As it can be seen in Figure 4, at pH 8 approximately 45% of the linked drug was released from the polymeric conjugate within 24 hours in the presence of esterase, while less than 12% was released in the absence of the enzyme. In addition, the structure of the released drug was verified. The molecular ion at m/z = 456 and the fragment at m/z = 438, due to the loss of water, confirmed the presence of ursolic acid. These results show the capacity of mPEG-UA to release free drug on the basis of the hydrolytic activity of esterase.

**Fig. 5:**
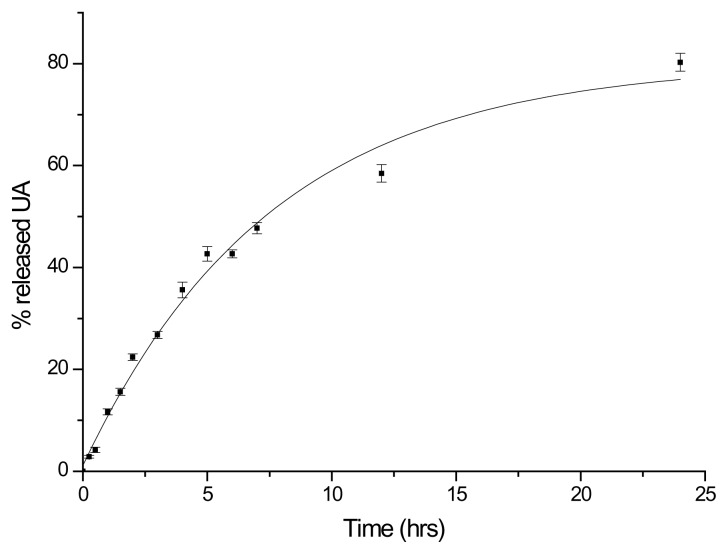
Release of UA ■ from mPEG-UA in human plasma at 37±0.1°C. Each experiment was carried out in triplicate and expressed as the mean value ± S.D.

The macromolecular prodrug’s ability to release free drug was also evaluated in human plasma. More than 78% of linked UA was released from the polymeric conjugate within 24 hours (Figure 5). This indicates the possibility of this macromolecular conjugate of constituting a good substrate for the plasma enzymatic complex and its capacity to deliver free and active UA in a prolonged manner. In addition, the structure of released UA was confirmed by mass spectrometry analysis.

In conclusion, in this paper the synthesis of a polymeric UA conjugate was described. UA was covalently linked to the activated macromolecular carrier, monomethoxypoly(ethylene glycol), via an intermediate ester formation. The simplicity, sensitivity, and rapidity of this synthesis allow it to be easily adapted for prodrug use. The PEG-UA prodrug showed some interesting peculiarities which make it attractive in the drug delivery for various treatments. It was very soluble in water and stable in physiological buffer, but in plasma, it was able to release the drug in a constant and effective manner.

## Experimental

### Materials and Methods

mPEG-OH (MW = 5,000 Da), dimethylaminopyridine (DMAP), 1,3-diaminopropane, *N*,*N*′-disuccinimidyl carbonate (DSC), *N*-(3-dimethylaminopropyl)-*N*′-ethylcarbodiimide (EDC), and 1-hydroxybenzotriazole (HOBT) were purchased from Fluka (Buchs, Switzerland). Ursolic acid (UA) and esterase from porcine liver – ammonium sulfate suspension, ≥150 units/mg protein (biuret) – was purchased from Sigma (St. Louis, Mo, USA). Other reagents (analytical grade) were bought from commercial suppliers and used without further purification unless otherwise noted.

The synthesis of mPEG-*linker*-NH_2_ (M.W. = 5,000 Da of mPEG) was carried out by following the procedures previously described [8].

### Analytical Methods

HPLC analyses were carried out with a Perkin-Elmer HPLC connected to a variable-wavelength UV detector. RP-HPLC analysis for UA was carried out according to the method previously described by Zacchigna *et al.* [2]. A LiChrosorb RP18 column (5 μm, 250 mm × 4.6 mm i.d., Perkin-Elmer) was used. A mixture of methanol–water–tetrahydrofuran (94:5:1 v/v), pH 5 was employed as the mobile phase, the detection wavelength was 220 nm, and the flow rate was 1 mL/min.

Quantification was performed on the basis of a linear calibration plot of peak area against concentration. Each curve was based on seven concentrations of the standard. Identification of UA was made by the comparison of the retention times with those of pure standard.

The amount of free NH_2_ was calculated using the colorimetric TNBS test [9]. The TNBS analysis was made on 2 mg samples of mPEG-NH_2_, accurately weighted, and added to a 10 ml flask with 9 ml of borate buffer 0.1 M (pH 9.3) and with 0.25 ml of a 0.03 M solution of TNBS in the same buffer. The volume was adjusted to 10 ml and after 30 min the absorbance at 421 nm (ɛ = 12850) gave the amount of free NH_2_ groups.

The ^1^H NMR spectra were recorded on a Jeol Ex 400 spectrometer (400 MHz). Chemical shifts were given in ppm from TMS as the internal standard.

FAB-MS was recorded on the Kratos MS 80 RFA mass spectrometer using a beam of Argon/Xenon (2–8), using methanol as a solvent and glycerol as a matrix.

The purity of the synthesized compounds was checked by TLC on pre-coated alumina, aluminium sheets (Merck 60 F254). Spots were visualized under 254 nm illumination.

### Synthesis of mPEG_5000_-O-CO-NH-CH_2_-CH_2_-CH_2_-NH-succ-UA (mPEG-UA)

#### Ursolic Succinate [2]

Ursolic acid [UA] **1** (1 g, 2.19 mmol) was dissolved in THF, (50 mL) and 1.2 equivalents of succinic anhydride (263 mg, 2.63 mmol), 0.25 equivalents of DMAP, and TEA (307 μL) were added. The mixture was stirred at room temperature for an appropriate time. The progress of the reaction was followed by TLC. When the reaction was complete, the solvent was concentrated under vacuum in a rotating flask and the solids were dispersed in water, then acidified to pH 3–4 with HCl, filtered, washed with water to neutrality, co-evaporated from anhydrous ACN, and gave a white solid. Product **2** was recrystallized from EtOH/H_2_O; 9:1 and dried over KOH under vacuum. Yield 88%.

TLC: CH_2_Cl_2_/MeOH 9:1 v/v, R_f_ UA = 0.64; R_f_ UA succinate = 0.71.

^1^H NMR (C_5_D_5_N): δ = 0.88 ppm (d; 1H, H-5), 0,92 (s; 3H, H_3_-25), 0.98 (s; 3H, H_3_-30), 1.02 (s; 3H, H_3_-29), 1.03 (s; 3H, H_3_-24), 1.07 (m; 1H, H-20), 1.08 (s; 3H, H_3_-26), 1.10, 1.59 (d; 2H, H_2_-1), 1.14, 2.36 (m; 2H, H_2_-15), 1.25 (s; 3H, H_3_-23), 1.25 (s; 3H, H_3_-27), 1.40, 1.49 (m; 2H, H_2_-11), 1.49 (m; 1H, H-19), 1.59, 1.40 (m; 2H, H_2_-6), 1.60, 1.40 (m; 2H, H_2_-7), 1.66 (t; 1H, H-9), 1.83 (m; 2H, H_2_-2), 1.97 (m; 2H, H_2_-11), 1.98 (m; 2H, H_2_-22), 2.13, 2.03 (m; 2H, H-12), 2.52 (t; 2H, CH_2_COO-UA), 2.65 (d; 1H, H-18), 2.83 (t; 2H, CH_2_CH_2_ H), 3.87 (dd; 1H, H-3), 5.52 (s; 1H, H-12).

EI-MS (70 eV): m/z: 556 [M, 15%]^+^, 497 [M-HO_2_CCH_2_, 30%]^+^, 455 [M-COCH_2_CH_2_CO_2_H, 48%]^+^, 438 [M-C_4_H_6_O_4_, 100%]^+^, 423 [M-C_4_H_6_O_4_, 18%]^+^, 408 [M-C_4_H_6_O_4_-2CH_3_, 25%]^+^, 393 [M-C_4_H_6_O_4_-3CH_3_, 28%]^+^.

#### mPEG-linker-NH_2_ [3]

The synthesis of mPEG-O-CO-NH-CH_2_-CH_2_-CH_2_-NH_2_ was carried out following the procedures previously described [8].

TNBS test: 0.98 moles of NH_2_ per mole of mPEG_5000_-O-CO-NH-CH_2_-CH_2_-CH_2_-NH_2._

#### mPEG-linker-ursolic [mPEG-UA] [4]

mPEG*-linker*-NH_2_ **3** (0.099 mmol, 0.495 g) was co-evaporated from anhydrous ACN and dried for 30 min under vacuum. The residue was dissolved in anhydrous ACN (10 mL). An ice solution of UA succinate **2** (0.297 mmol; 0.164 g), HOBT (0.297 mmol; 40.12 mg), EDC (0.297 mmol; 57 mg), and TEA (0.297 mmol; 42 μl) in anhydrous ACN (10 mL) was added very slowly dropwise under stirring to the solution of mPEG-NH_2_. Once the addition was completed, stirring was continued at room temperature overnight. The solvent was evaporated and the residue taken in CH_2_Cl_2_, filtered, precipitated by slow addition under stirring on an ice bath of anhydrous diethyl ether, recovered by filtration and washed with anhydrous diethyl ether. The product was recrystallized from EtOH (100 mL) and dried over KOH under vacuum. Yield 95%.

^1^H NMR (DMSO-d6): δ = 0.89 ppm (d; 1H, H-5), 0.92 (s; 3H, H_3_-25), 0.99 (s; 3H, H_3_-30), 1.05 (s; 3H, H_3_-24), 1.05 (s; 3H, H_3_-29), 1.08 (m; 1H, H-20), 1.09 (s, 3H, H_3_-26), 112, 1.60 (d; 2H, H_2_-1), 1.25, 2.38 (m; 2H, H_2_-15), 1.26 (s; 3H, H_3_-23), 1.26 (s; 3H, H_3_-27), 1.41, 1.50 (m; 2H, H_2_-21), 1.48 (m; 1H, H-19), 1.51-1.40 (m; 2H, -NH-CH_2_CH_2_ CH_2_ NH), 1.60, 1.39 (m; 2H, H_2_-6), 1.62, 1.42 (m; 2H, H_2_-7), 1.69 (t, 1H, H-9), 1.84 (m; 2H, H_2_-2), 1.98 (m; 2H, H_2_-11), 2.0 (m; 2H, H_2_-22), 2.14, 2.06 (m; 2H, H_2_-16), 2.53 (t; 2H, CH_2_COO-UA), 2.66 (d; 1H, H-20), 2.85 (t; 2H, CH_2_CONH), 3.25-2.80 (m; 4H, -NH-CH_2_CH_2_CH_2_NH-), 3.75-3.36 [brs, 452.9 CH_2_CH_2_O (PEG)], 3.86 (dd; 1H, N-3), 4.03 (brs; 2H, mPEG-CH_2_OCO), 5.52 (s; 1H, H-12), 7.90 (brs, NH).

The absence of the free drug in the adduct was confirmed by HPLC analysis.

The content of linked UA in the conjugate was measured by HPLC analysis on the basis of the release of UA in alkaline media after 15 min at 80°C. This was found to be 100% (w/w).

### Drug Release Studies

#### Buffer Solution Hydrolysis

To investigate the hydrolytic stability of mPEG-UA, 10 mg/mL of **4** were dissolved in two different buffers, HCl/NaCl/glycine buffer, pH 1.2, 0.2 M, and a phosphate buffer, pH 7.4, 0.1 M, each containing an appropriate quantity of internal standard, at 37±0.1°C. Samples were taken at suitable intervals and the quantity of UA released by hydrolysis was quantified by the HPLC method. Every experiment was repeated in triplicate.

#### Enzymatic Hydrolysis

The hydrolytic stability of the drug-polymer linkage of the polymeric conjugate to esterase was assessed in 0.08 M Tris buffer, 0.1 M CaCl_2_ at pH 8 with an adduct 1×10^−4^ M concentration in UA. Samples of 1 mL of mPEG-UA solution were added to 100 μL of a porcine esterase solution. The solutions were incubated at 37±0.1°C and sampled at suitable time intervals by HPLC and EI-MS analysis. As a control, analogous experiments were performed by adding the buffer solution without the enzyme to the conjugate solutions. Every experiment was repeated in triplicate.

EI-MS (70 eV) : m/z = 456 [M, 10%]^+^, 438 [M-H_2_O, 22%]^+^, 248 [M-C_7_H_13_O_2_-H_2_O-4CH_3_, 88%]^+^, 233 [10%]^+^, 219 [25%]^+^, 203 [67%]^+^, 189 [22%]^+^, 133 [100%]^+^, 119 [20%]^+^.

#### Plasmatic Hydrolysis

The hydrolysis of the mPEG-UA conjugate was studied in human plasma at 37±0.1°C. The human blood (1.5 ml) from three donors was distributed in polystyrene tubes and centrifuged at 1000 rpm (225 ×g) for 10 minutes in a Eppendorf Centrifuge mod. 5415 at 25°C. The supernatant was removed from each tube, pooled, and centrifuged again at 1000 rpm for 10 minutes to eliminate the remaining erythrocytes and leukocytes. The plasma was stored in a freezer at −80°C for the pending assay [11]. The reactions were initiated by adding 100 μL of the aqueous solution of the adduct (10 mg/mL) to samples (1 mL) of preheated plasma. At scheduled times, the samples were analyzed after solid-phase extraction (SPE) by HPLC and EI-MS analysis. Every experiment was repeated in triplicate.

EI-MS (70 eV) : m/z = 456 [M, 10%]^+^, 438 [M-H_2_O, 22%]^+^, 248 [M-C_7_H_13_O_2_-H_2_O-4CH_3_, 88%]^+^, 233 [10%]^+^, 219 [25%]^+^, 203 [67%]^+^, 189 [22%]^+^, 133 [100%]^+^, 119 [20%]^+^.

#### Extraction Procedure

The plasma samples were extracted using a solid-phase extraction technique. In brief, 1 ml of plasma was vortexed for 30 seconds. Subsequently, samples were applied into the RP Supelclean^®^ LC18 (Supelco, Bellefonte, PA, USA), pre-treated with 1 mL reservoir volume (RV) methanol followed by 1 RV water. The LC18 cartridges were placed on a Visiprep^®^ sample processing station and subsequently washed twice with 1 RV water to remove the water-soluble fractions (mPEG-linker-NH_2_, mPEG-UA) under vacuum. The UA was eluted with 1 mL methanol. The eluent was evaporated under a stream of nitrogen at 40°C using the TurboVab LV evaporator (Zymak, Hopkinton, MA, USA), and the dry residue was redissolved in 50 μL MeOH. Subsequently, samples were centrifuged for 10 minutes at 10000 g and 20 μL aliquots were used for HPLC analysis.
